# Can large language models assist with pediatric dosing accuracy?

**DOI:** 10.1038/s41390-025-03980-8

**Published:** 2025-03-08

**Authors:** Chedva Levin, Brurya Orkaby, Erika Kerner, Mor Saban

**Affiliations:** 1 Faculty of School of Life and Health Sciences, Nursing Department, The Jerusalem College of Technology-Lev Academic Center, Jerusalem, Israel; 2 The Department of Vascular Surgery, The Chaim Sheba Medical Center, Tel Hashomer, Ramat Gan, Tel Aviv Israel; 3 The Department of Hemodialysis children, Shaare Zedek Medical Center, Jerusalem, Israel; 4 Department of Nursing Sciences, School of Health Professions, Faculty of Medical and health Sciences, Tel Aviv University, Tel Aviv, Israel

## Abstract

**Background and Objective:**

Medication errors in pediatric care remain a significant healthcare challenge despite technological advancements, necessitating innovative approaches. This study aims to evaluate Large Language Models’ (LLMs) potential in reducing pediatric medication dosage calculation errors compared to experienced nurses.

**Methods:**

This cross-sectional study (June-August 2024) involved 101 nurses from pediatric and neonatal departments and three LLMs (ChatGPT-4o, Claude-3.0, Llama 3 8B). Participants completed a nine-question survey on pediatric medication calculations. Primary outcomes were accuracy and response time. Secondary measures included seniority and group membership on accuracy.

**Results:**

Significant differences (*P* < 0.001) were observed between nurses and LLMs. Nurses averaged 93.14 ± 9.39 accuracy. Claude-3.0 and ChatGPT-4o achieved 100 accuracy, while Llama 3 8B was 66 accurate. LLMs were faster (15.7–75.12 seconds) than nurses (1621.2 ± 8379.3 s).

The Generalized Linear Model analysis revealed task performance was significantly influenced by duration (Wald χ² = 27,881.261, *p* < 0.001) and interaction between relative seniority and group membership (Wald χ² = 3,938.250, *p* < 0.001), with participants achieving a mean total grade of 91.03 (SD = 13.87).

**Conclusions:**

Claude-3.0 and ChatGPT-4o demonstrated perfect accuracy and rapid calculation capabilities, showing promise in reducing pediatric medication dosage errors. Further research is needed to explore their integration into practice.

**Impact:**

- **Key Message** Large Language Models (LLMs) like ChatGPT-4o and Claude-3.0 demonstrate perfect accuracy and significantly faster response times in pediatric medication dosage calculations, showing potential to reduce errors and save time.
- **Addition to Existing Literature** This study provides novel insights by quantitatively comparing LLM performance with experienced nurses, contributing to the understanding of AI’s role in improving medication safety.
- **Impact** The findings emphasize the value of LLMs as supplemental tools in healthcare, particularly in high-stakes pediatric care, where they can reduce calculation errors and improve clinical efficiency.

## Introduction

Medical errors remain a significant issue in healthcare, with medication errors being a leading cause of preventable harm. In their landmark report “To Err Is Human”, published in 2000, the Institute of Medicine estimated that between 44,000 to 98,000 people die in U.S. hospitals each year due to medical errors.^1^ A subsequent report in 2001, “Crossing the Quality Chasm,” highlighted the critical need to improve patient safety and quality of care.^2^

Particularly concerning are errors related to the administration of medications, which often stem from mistakes in calculating the correct dosage, especially for pediatric patients. Children are at higher risk for medication errors due to the complexities of weight-based dosing.^3,4^ Despite the implementation of technologies such as computerized physician order entry (CPOE) and clinical decision support systems (CDSS), which aim to minimize such errors, studies have shown that medication calculation errors persist.^5,6^ A systematic review conducted by Wong et al. in 2012 concluded that, out of the 16 studies analysed, dosing errors constituted the most common type of medication error, highlighting a pressing need for further innovation in this critical area.^7^

Recent studies have shown that errors in drug dosing for pediatric patients are alarmingly prevalent. A 2022 review highlighted that pediatric patients experience medication errors at a rate of 31%, significantly higher than the 13% observed in adults, with millions of preventable errors occurring in this population.^8^ Moreover, Takata et al. identified that 22% of drug related events in pediatric patients could have been prevented.^9^

Although technologies such as electronic health records (EHRs) have been implemented to mitigate these mistakes, ongoing research continues to reveal that errors remain prevalent.^10^ The literature suggests that while these systems can enhance accuracy, they often rely on the clinician’s input, which can result in errors.^11^ In the field of healthcare, Large Language Models (LLMs) have shown promise in various applications, including clinical documentation and decision support.^12–14^ A 2023 study indicated that language models can assist with patient diagnosis.^15^ While another study reported improved patient outcomes attributed to the use of LMMs.^16^ Furthermore, a 2024 study found that LLMs can provide crucial clinical decision support, particularly in the fields of oncology, radiology and dermatology.^17^ Another study from 2024 revealed that integrating language models with healthcare professionals can significantly reduce drug related problems^18^

The rationale for this study lies in addressing the persistent gap in pediatric medication dosing accuracy. LLMs offer a unique advantage by rapidly retrieving evidence-based dosing guidelines, cross-referencing complex data points, and minimizing the cognitive burden on healthcare professionals. Unlike traditional CDSS tools, LLMs are capable of contextual reasoning, enabling them to provide tailored recommendations based on patient-specific variables. By leveraging the power of LLMs, this research paper aims to address the critical issue of medication dosage calculation and administration errors in pediatric care by evaluating the potential contribution of LLMs in enhancing accuracy and reducing errors. Specifically, the study compares the performance of LLMs with experienced nurses in calculating and administering pediatric drug doses, highlighting the strengths and limitations of this technology in a high-stakes clinical context.

## Methods

### Design

This cross-sectional study was conducted over a three-month period from June to August 2024. The research utilized a survey instrument comprising nine validated questions focused on medication calculations for pediatric drug administration.

### Participants

The study included four distinct groups of participants. The first group consisted of nurses working in various pediatric and neonatal departments, including inpatient departments, intensive care units, emergency medicine departments, day hospitalization units, and centers for children with chronic kidney diseases or other chronic conditions.

Nurses were recruited voluntarily from multiple healthcare centers to ensure a diverse sample encompassing varying levels of experience, unit roles, and clinical backgrounds. Recruitment was conducted through institutional announcements and invitations shared within nursing departments, aiming to capture a representative range of participants. While efforts were made to include nurses from varied settings, we acknowledge that the sample may not fully represent the broader nursing population. Seniority in this study was defined by years of clinical experience rather than specific roles or titles within nursing units. This approach was chosen to standardize comparisons across participants and minimize variability arising from differing organizational role structures. For evaluating performance, a binary scoring system was applied: correct responses were assigned a score of 100, while incorrect responses received a score of 0. This method provided a clear and objective assessment metric, though we recognize it may not fully capture the reasoning behind certain responses. Initially, a detailed classification scheme was proposed to categorize nurses by experience levels and roles. However, to enhance clarity and ensure consistency in analysis, this classification was streamlined to focus primarily on years of clinical experience. These methodological decisions were made to improve transparency, simplify interpretation, and align with the study’s objectives while addressing the nuances of nurse performance evaluation.

The second, third, and fourth groups were the LLMs ChatGPT-4o, Claude-3.0, and LLAMA 3 8B, respectively.

The inclusion criteria for nurses were: (1) Being a registered nurse (2) Holding a valid nursing license in their state/province of practice (3) Being employed in pediatric and neonatal departments during the data collection period (4) Providing informed consent to participate in the study.

### Data collection

Sociodemographic data collected from the nurses included sex, age, years of seniority, type of pediatric department, academic status, and basic training.

### Study questionnaire and Procedure

The study involved nine questions related to pediatric medication dosage calculations, categorized into two main types:

1. **Dose Calculations Based on the Child’s Weight or Drug Administration Rate:**

- Example: *‘On a doctor’s order, a child weighing 10* *kg should be given Dipyrone 10* *mg/kg. The bottle contains Dipyrone 1.25* *g/5* *ml, the bottle contains 50* *ml. How many ml of medicine should be given?’*

2. **Dose Calculations Based on Product Concentration:**

- Example: *‘An instruction was given to give IBUPROFEN 300* *mg in syrup. The bottle says 100* *mg/5* *ml. How many ml should be given?’*

The survey questions were collaboratively developed by the two authors, both senior nurses with over 25 years of clinical experience and doctoral qualifications. Their expertise is complementary: one specializes in pediatric ward care, while the other focuses on intensive care. The questions were meticulously reviewed to align with established clinical protocols, ensuring clarity, relevance, and representation of real-world challenges in pediatric medication dosing. Nurses submitted their responses to the survey using Qualtrics XM, an online survey tool (Pediatric nurses’ questionnaire are appear in Appendix 1).

Concurrently, the models ChatGPT-4o, Claude-3.0, and Llama 3 8B were tasked with solving the same nine questions to evaluate their accuracy and reliability.

*ChatGPT-4o*, developed by OpenAI, is a multimodal large language model known for its advanced natural language processing capabilities and ability to handle complex tasks.

*Claude-3.0*, created by Anthropic, is designed to engage in nuanced dialogue and perform a wide range of language tasks with high accuracy.

*Llama 3 8B*, developed by Meta AI, is an open-source large language model that has shown promising performance in various language understanding and generation tasks. These models were selected for their diverse architectures and training approaches, allowing for a comprehensive evaluation of LLMs capabilities in pediatric medication dosage calculations.

The specific prompt used for all LLMs in this study was: *“Calculate the correct pediatric medication dosage based on the provided weight, age, and drug guidelines.”* This prompt was kept consistent across all models to ensure comparability in responses.^19^ The responses from these models were collected and analyzed alongside the nurses’ submissions, providing insights into the potential of LLMs as a tool for mitigating medication dosing errors in pediatric care. See an Example of LLMs calculation processes in Appendix 2.

### Data analysis

Participant responses were binary coded: correct answers received a score of 100, while incorrect answers were assigned a score of 0. Response times were recorded for both human participants (nurses) and LLMs, measured from the presentation of question details to the generation of the final response. Mean response times were calculated across all questions for each system.

Two levels of seniority were calculated for each participant:

1) Basic Seniority: For nurses, this was determined by the number of months spent in nursing studies. For LLMs, it was calculated from the date they were first deployed on the network; 2) Relative Seniority: For nurses, this was determined by their actual professional experience. For LLMs, it was calculated as the number of months since the release of the specific version used in the present study.

Statistical methods were selected based on the nature of the data. For continuous variables, means and standard deviations were calculated. Categorical variables were summarized using frequencies and percentages. ANOVA was employed to compare means across groups where appropriate.

A Generalized Linear Model (GLM) analysis was performed to predict task performance, with results considered significant at *p* < .001, incorporating duration, basic seniority, and the interaction between relative seniority and group membership as predictors.

All statistical analyses were performed using SPSS Version 28 software.

### Ethical considerations

The university’s ethics committee granted approval for this study (reference number #0006223-2) prior to its commencement. Throughout the data collection process, we ensured participant anonymity. All participating nurses provided informed consent and were made aware of their right to discontinue their involvement at any point, without needing to provide a reason.

## Results

The study involved 101 nurses from pediatric and neonatal departments, with 25 (24.8%) holding master’s degrees. Participants were distributed across inpatient wards: 41 (40.6%) in pediatric hospitalization, 30 (29.7%) in pediatric intensive care, 11 (10.9%) in emergency medicine, 8 (7.9%) in day hospitalization for children, and 11 (10.9%) in chronic care settings for children. Most nurses (64.4%) had advanced training beyond basic qualifications. The sample’s mean age was 37.4 ± 9.68 years (range: 24–63), with 95% being women (Table 1).

**Table 1:** Sociodemographic characteristics of the study sample (*N* = 101).

Variable	Range	Mean (SD)
Age	24–63	37.4 (9.68)
Seniority (year)	1–41	11.28 (9.82)
**Variable**		N (%)
Gender	Male	5 (5)
	Female	96 (95)
Academic Status	B. A	76 (75.2)
	M.A	25 (24.8)
Post basic course	Yes	65 (64.4)
Type of Inpatient department	Children’s department	41 (40.6)
	Pediatric/neonatal intensive care	30 (29.7)
	Pediatric Emergency Medicine	11(10.9)
	Children’s day hospitalization	8 (7.9)
	Medical centers for a chronically ill child	11(10.9)

Nurses’ basic seniority averaged 32 months, compared to 22 months for ChatGPT-4o, 17 months for Claude-3.0, and 18 months for Llama 3 8B. Relative seniority for nurses was 134.6 ± 118.25 months, while for AI models it was 3 months (ChatGPT-4o), 2 months (Claude-3.0), and 4 months (Llama 3 8B). Mean response times were 1621.2 ± 8379.3 seconds for nurses, and 26.22 seconds (ChatGPT-4o), 75.12 seconds (Claude-3.0), and 15.7 seconds (Llama 3 8B) for the LLMs.

From Table 2, we can observe that significant differences (*P* < 0.001) were observed between nurses and LLMs in their responses to questions 2, 3, 4, 5, and 8. For questions 6 and 9, the differences were also significant, albeit at a lower threshold (*P* < 0.05). Question 7 showed a borderline difference (*P* = 0.05). Only for question 1 was there no significant difference between the nurses’ responses and the models’ responses.

**Table 2:** Differences in the scores of the nurses compared to the large language models.

Mean (SD) (range 0–100)	Nurse	Claude 3.5	ChatGPT-4.0	LLAMA 3 8b	F score	*P*-value
**Question 1**	98.99 (10.05)	100	100	100	0.86	0.46
**Question 2**	91.09 (28.63)	100	100	0	804.3	**0.00**
**Question 3**	98.0 (14.07)	100	100	0	3495.1	**0.00**
**Question 4**	93.07 (25.52)	100	100	0	1023.1	**0.00**
**Question 5**	83.17 (37.60)	100	100	100	17.07	**0.00**
**Question 6**	96.04 (19.60)	100	100	100	3.47	**0.01**
**Question 7**	97.03 (17.06)	100	100	100	2.58	0.05
**Question 8**	85.0 (35.88)	100	100	100	14.89	**0.00**
**Question 9**	96.94 (17.31)	100	100	100	2.66	**0.04**
**Total Questions**	93.14 (9.39)	100	100	66.66	793.6	**0.00**

The bold values indicate statistically significant p values (*p* < 0.05), suggesting a meaningful difference between the scores for these questions.

The Table 3 presents the results of a Generalized Linear Model analysis examining task performance across groups, based on data from 356 participants. The analysis used Total Grade (The average score of 9 research questions).

**Table 3:** Generalized Linear Model Analysis of Task Performance Across Groups (*N* = 356).

Parameter	Value	Test Statistics
**Model Information**		
Dependent Variable	Total Grade	
Probability Distribution	Normal	
Link Function	Identity	
**Model Fit**		
Deviance/df	0.243	
Log Likelihood	−193.253	
AIC	592.506	
BIC	991.624	
**Type III Tests**	Wald χ²	*p*-value
Intercept	8,972,964.143	< 0.001
Duration (seconds)	27,881.261	< 0.001
Relative seniority × GROUP	3,938.250	< 0.001
Basic seniority	NC^a^	-
**Descriptive Statistics**		
Mean Total Grade (SD)	91.03 (13.87)	
Range	55.56–100.00	

*AIC* Akaike’s Information Criterion, *BIC* Bayesian Information Criterion, *NC*^*a*^ Not computed due to numerical problems, *SD* Standard Deviation. Model includes interaction effects between relative seniority and group membership.

The model fit statistics indicate a relatively good fit, with a deviance per degree of freedom of 0.243 and a log likelihood of −193.253. The model’s information criteria (AIC = 592.506, BIC = 991.624) provide measures of model quality while accounting for model complexity.

The Type III Tests revealed several significant effects: The intercept was highly significant (Wald χ² = 8,972,964.143, *p* < 0.001) Duration in seconds showed a strong effect (Wald χ² = 27,881.261, *p* < 0.001). The interaction between relative seniority (in months) and group membership (nurses vs. LLMs), was significant (Wald χ² = 3,938.250, *p* < 0.001).

Descriptive statistics show that participants achieved a mean total grade of 91.03 (SD = 13.87), with scores ranging from 55.56 to 100.00, indicating generally high performance with some variability.

**Fig. 1:**
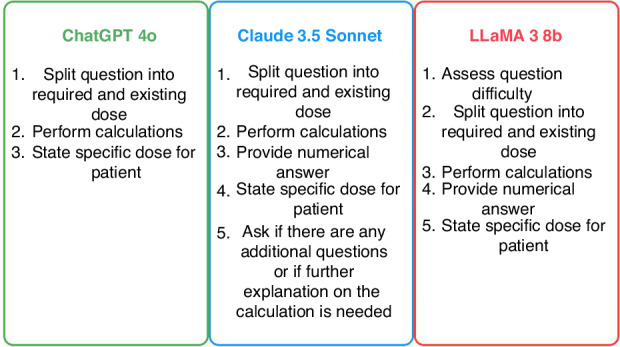
GenAI approaches to medication calculation.

Figure 1 illustrates the step-by-step calculation processes employed by the three LLMs in medication dosage calculations. ChatGPT-4o uses a streamlined three-step approach, focusing on splitting the question, performing calculations, and stating the specific dose. Claude 3.5 Sonnet follows a more detailed five-step process, adding a numerical answer step and offering further explanation if needed. Llama 3 8B uniquely begins by assessing question difficulty before following a similar process to Claude, but without the offer for additional explanation. These differences highlight the varying strategies and levels of user engagement among LLMs in addressing complex medical calculations.

## Discussion

This study comparing the performance of nurses and various LLMs in pediatric medication dosage calculations provides critical insights into the potential role of LLMs in mitigating medication errors, a persistent challenge in pediatric care. The findings reveal significant variations in performance among different LLMs, highlighting the importance of distinguishing between individual models rather than treating all LLMs as a homogeneous group.

Two models, ChatGPT-4o and Claude 3.5, demonstrated exceptional accuracy, correctly answering 100% of the questions. This performance suggests promising capabilities for these specific LLMs in supporting healthcare professionals with complex calculations. However, it’s crucial to note that not all LLMs performed equally well. For instance, Llama 3 8B made errors in approximately one-third of its calculations, all of which were reduction errors. In the context of pediatric medication dosing, such discrepancies could have severe consequences, underscoring the critical nature of accuracy in this domain.^20^

In the case of Llama 3 8B, the identification of consistent reduction errors points to a specific weakness in its calculation process for pediatric dosages. This finding is crucial for understanding the limitations of certain LLMs and the potential risks associated with their use in critical healthcare applications. It underscores the importance of model-specific evaluation rather than generalizing capabilities across all LLMs.^21^

The significant differences observed between nurses and LLMs in their responses to most medication calculation questions highlight the complexity of this task and the varying strengths of human and LLMs approaches. These results align with recent studies exploring the capabilities of LLMs in healthcare. Yang et al.^22^ emphasized the potential of LLMs in various healthcare applications, including clinical decision support and medical education. Our study extends this line of inquiry specifically into pediatric medication dosing, where errors can have severe consequences. While our research didn’t delve into the nature of errors for all LLMs (as ChatGPT-4o and Claude 3.5 were 100% accurate), it did highlight an important distinction between consistent and systematic errors in the context of LLAMA’s performance^23^ The performance disparities among LLMs underscore the complexities inherent in their design and application. Reduction errors in LLMs often stem from limitations in model capacity, training data quality, and the intricacies of the tasks they are applied to. For instance, in benchmarks like the MMLU (Massive Multitask Language Understanding), Llama 3 8B achieved a micro average score of 67.4%, indicating challenges in handling diverse and complex queries with high accuracy.^24^

A significant area of concern for Llama 3 8B is its handling of logical reasoning tasks. Studies have demonstrated that it struggles with simple reasoning problems, often providing incorrect answers with high confidence. This issue is exemplified in tasks like the “Alice in Wonderland problem,” where the model failed to deduce the correct number of sisters based on given sibling information, highlighting a breakdown in basic reasoning capabilities.^25^

Generalized Linear Model indicate a relatively good fit, with a deviance per degree of freedom of 0.243 and a log likelihood of −193.253. The intercept was highly significant (Wald χ² = 8,972,964.143, *p* < 0.001) Duration in seconds showed a strong effect (Wald χ² = 27,881.261, *p* < 0.001). The interaction between relative seniority (in months) and group membership (nurses vs. LLMs), was significant (Wald χ^2^ = 3,938.250, *p* < 0.001). This finding emphasizes the importance of experience in nursing that provides skills and strategies to improve abilities for drug calculations or the importance initial LLMs development.^26,27^

The stark contrast in response times between nurses (mean 1621.2 ± 8379.3 seconds) and LLMs (15.7–75.12 s) raises important questions about the potential role of LLMs in time-sensitive clinical situations.^27,28^

The step-by-step calculation processes illustrated by the LLMs provide valuable insights into their problem-solving approaches. This transparency aligns with the emphasis on explainability in AI models used in healthcare, as highlighted by Yu et al.^29^ Their comprehensive roadmap for healthcare integration of AI emphasizes the need for explainability, interdisciplinary collaboration, ethical considerations, and continuous evaluation. Our study contributes to this roadmap by providing concrete examples of LLMs reasoning in a critical healthcare task, which could be beneficial for educational purposes and for validating AI-generated calculations^30,31^ However, it also raises questions about the ability of LLMs to consider nuanced, patient-specific factors that experienced nurses might take into account.

Integrating LLMs with Electronic Medical Records (EMRs) offers a promising avenue to enhance pediatric dosing accuracy. LLMs can process vast amounts of unstructured data, providing context-aware recommendations that complement the structured data within EMRs. This synergy can streamline clinical workflows, reduce cognitive load on healthcare providers, and potentially improve patient outcomes.

While LLMs offer significant potential in healthcare, particularly in pediatric medication dosing, several challenges must be addressed to ensure their effective integration. Experienced nurses often intuitively assess nuanced, patient-specific factors—such as subtle clinical signs, psychosocial elements, and individual patient preferences—that LLMs may not fully capture. Additionally, incorporating LLMs into existing EMR systems presents technical challenges, including interoperability issues and the necessity for user training to facilitate seamless adoption. Ethical considerations, such as data privacy and the risk of over-reliance on AI-generated recommendations, are also critical to maintaining the integrity of patient care. Therefore, integrating LLMs into healthcare requires careful consideration and thorough evaluation to ensure safety and efficacy. Our empirical evidence on the performance of LLMs in this specific healthcare task showcases the potential of AI in pharmaceutical calculations. These findings contribute significantly to the growing body of research on the integration of LLMs in healthcare settings.

A key finding of our study is the strong performance of two LLM models, ChatGPT-4o and Claude 3.5, which achieved 100% accuracy in pediatric medication dosage calculations within the scope of our study. This high level of accuracy highlights the potential for artificial intelligence to enhance pharmaceutical calculations. Unlike clinical decision-making scenarios that involve complex, evolving cases, pharmaceutical calculations are typically focused tasks based on specific medical instructions and patient data. In this context, the AI model’s role is primarily to solve a mathematical problem rather than interpret nuanced clinical information. However, it is important to note that while these results are promising, extensive validation across a larger dataset with diverse and complex scenarios is necessary before LLMs can be considered reliable for widespread clinical application. These findings contribute significantly to the growing body of research on the integration of LLMs in healthcare settings.

Finaly, our research underscores the need for rigorous testing, validation, and careful implementation of any AI-based systems in critical healthcare applications, particularly those involving pediatric care. The variations in performance and levels of explainability among different LLMs emphasize the importance of model-specific evaluation. This study also highlights the indispensable value of human expertise in the implementation of nursing work.

In line with Yu et al.‘s roadmap, our findings support the need for continued interdisciplinary collaboration in the development and implementation of AI in healthcare. As we move forward, it will be crucial to maintain a balance between leveraging the potential of AI technologies and ensuring patient safety through human oversight and expertise. Future research should continue to explore the integration of LLMs in various healthcare tasks, always with a focus on ethical considerations, patient safety, and the complementary roles of AI and human healthcare professionals.

### Limitations

This study has several limitations. First, the static nature of the AI models used, which may not reflect the rapid advancements in AI technology. Additionally, while our questions were validated and developed by experienced professionals, they may not fully capture the complexity of real-world practice, which often involve patient-specific factors beyond simple calculations. Third, the calculation of pharmacists at work is sometimes affected by the environment, including noise, work pressure, or phone calls, which make concentration difficult and lead to calculation errors. In the current study, in some cases, the nurses responded in an environment that is not similar to the natural work environment, which may affect the results. Also, while the nine questions evaluated in this study were carefully selected to represent common pediatric dosing scenarios, they do not encompass the full variability encountered in real-world clinical practice. LLMs, such as GPT-4, have been trained on diverse datasets, enabling them to process a wide range of clinical questions and adapt to novel scenarios. However, further research is needed to assess their performance across a broader array of clinical cases, including complex, edge-case dosing scenarios and ambiguous instructions. Future studies should focus on evaluating LLMs’ adaptability and robustness in dynamic, real-time clinical environments to better understand their generalizability and practical utility in pediatric medication safety. Finally, when research is conducted in non-clinical settings, it lacks the authentic stressors present in clinical environments. This absence can result in data that do not accurately reflect nurses’ behaviors, decision-making processes, and coping mechanisms under typical work conditions.

### Conclusion and future direction

The integration of LLMs into healthcare presents significant opportunities to enhance patient care, particularly in critical areas like pediatric medication dosing. However, achieving 100% accuracy remains a formidable challenge. While LLMs have demonstrated impressive capabilities, their deployment in clinical settings must be approached with caution to ensure patient safety. Additionally, the integration of LLMs into clinical workflows poses challenges related to data privacy, ethical considerations, and the need for robust regulatory frameworks. Importantly, while LLMs excel at improving dosing calculation accuracy and reducing calculation time, they are not a substitute for human clinical judgment. LLMs cannot assess the appropriateness of the medication chosen, the route of administration, or patient-specific factors that require nuanced clinical evaluation. These aspects remain firmly within the domain of human expertise. Therefore, LLMs should be viewed as complementary tools designed to support, rather than replace, healthcare professionals in medication administration processes. This balanced integration of LLMs and human oversight is essential to ensure safe and effective outcomes in pediatric care.

To address these challenges, future research should focus on several key areas:

- **Longitudinal Assessments**: Evaluating how the performance of both nurses and LLMs evolves over time with additional training or updates is crucial. This includes assessing the impact of continuous learning and adaptation in dynamic clinical environments.
- **AI-Assisted Calculations in Real-World Settings**: Investigating the impact of AI-assisted calculations on overall medication error rates in real-world clinical settings will provide insights into the practical benefits and limitations of LLMs. Such studies can inform guidelines for effective AI integration into healthcare practices.
- **Complex Scenarios Involving Patient-Specific Factors**: Examining the performance of nurses and LLMs in complex scenarios that involve patient-specific factors and ethical considerations will offer a more comprehensive understanding of their respective strengths and limitations. This includes exploring how LLMs handle nuanced clinical situations that require contextual judgment.
- **Optimal Integration into Clinical Workflows**: Researching the optimal integration of AI tools into clinical workflows and their impact on healthcare provider work processes is essential. This involves understanding how AI can complement human expertise without disrupting established practices or introducing new risks.

As we advance the integration of LLMs in healthcare, it is vital to maintain a balanced perspective. While LLMs offer promising capabilities, they are not a substitute for human judgment. The future of pediatric medication safety likely lies in the thoughtful integration of LLMs to support and enhance human work processes, rather than replacing human expertise entirely.

In conclusion, while LLMs have the potential to significantly improve accuracy in medication dosage calculations, especially in high-stakes areas like pediatric care, their integration into clinical practice must be approached with careful consideration of the associated challenges. Ongoing research and collaboration between AI developers, healthcare professionals, and policymakers are essential to harness the benefits of LLMs while ensuring patient safety and ethical integrity.

## Supplementary information

Appendix 1

Appendix 2

## Data Availability

The data supporting the findings of this study are available from the corresponding author upon reasonable request. Due to privacy and ethical considerations, access to raw data will require appropriate permissions and adherence to institutional data-sharing agreements.
