# Supplementary material for: Can large language models assist with pediatric dosing accuracy?
Source: Pediatr Res. 2025 Mar 8;98(5):1760–5. doi: 10.1038/s41390-025-03980-8 (PMC12602315; doi:10.1038/s41390-025-03980-8)
Supplement: Supplementary file 1 — Appendix 1 [file 41390_2025_3980_MOESM1_ESM.pdf]

Appendix 1: Pediatric nurses questionnaire

1. An instruction was given to administer a fluid infusion of Normal Saline 0.9% 25 ml/hr for 24 hours. How many ml of fluids in total will the patient receive per day?
2. An order was received to administer 1000 units of Heparin in a push dose. On the ampoule is written Heparin 25000units=5ml, how many ml should be pumped?
3. You need to administer 350,000 units of penicillin to a patient. The penicillin bottle indicates a concentration of 500,000 units/2ml. How many ml should the patient receive?
4. On a doctor's order, a child weighing 5 kg should be given Vancomycin 20 mg/kg. The Vancomycin ampoule contains 500 mg and needs to be diluted in 10 ml of NaCl 0.9%. How many ml should you draw up to administer the correct dose?
5. An instruction was given to administer a suppository Nurofen 250mg PR. The Nurofen suppositories in the package are 125 mg each. How many suppositories should you give to the patient?
6. You need to give a patient IBUPROFEN 300 mg in syrup form. The bottle indicates a concentration of 100 mg/5 ml. How many ml should be given?
7. A 10 kg infant needs to receive Erythromycin 48 mg/kg per day, divided into 4 equal doses. How many mg of medication should the infant receive in each dose?
8. In the doctor's order for the medicine it is written: Tab DIGOXIN 0.125 mg*1/day. In the department there are tablets in 0.25 mg any tablet. How many tablets should be given to the patient per day?
9. On a doctor's order, a child weighing 10 kg should be given Dipyrone 10mg/kg. The bottle contains Dipyrone 1.25g/5ml, the bottle contains 50ml. How many ml of medicine should be given?
