# Supplementary material for: Can large language models assist with pediatric dosing accuracy?
Source: Pediatr Res. 2025 Mar 8;98(5):1760–5. doi: 10.1038/s41390-025-03980-8 (PMC12602315; doi:10.1038/s41390-025-03980-8)
Supplement: Supplementary file 2 — Appendix 2 [file 41390_2025_3980_MOESM2_ESM.pdf]

Appendix 2: Example of LLMs calculation processes

Question: You need to give a patient IBUPROFEN 300 mg in syrup form. The bottle indicates a concentration of 100 mg/5 ml. How many ml should be given?

ChatGPT

- **Split the question**
  - To calculate how many ml to give:
  - Drug concentration: 100 mg/5 ml = 20 mg/ml 300 mg are required
- **Perform calculations**
  - 300mg/20mg=15
- **Specific dose**
  - Give 15 ml

Claude 3.5 Sonnet

- **Split the question**
  - Let's solve this question step by step:
  - Given data:
    1. Required dosage: 300mg of IBUPROFEN
    2. Syrup concentration in bottle: 100mg/5ml
- **Perform calculations**
  - First, let's find how many ml contain 100mg:100mg = 5ml
  - Now, let's calculate how many ml are needed for 300mg
- **Provide numrical answer**
  - : If 100mg = 5ml, Then 300mg = x ml
  - We'll use a simple ratio: 300 / 100 = x / 5 x = (300 * 5) / 100 x = 1500 / 100 x = 15 ml
- **Specific dose**
  - You should give 15 ml of the syrup.
- **Offering further explanation**
  - Do you have any additional questions or would you like further explanation about the calculation?

Llama 3 8b

- **Assess question difficulty**
  - Very easy!
- **Split the question**
  - Instruction: IBUPROFEN 300mg in syrup.
  - The bottle shows 100mg/5ml.
- **Perform calculations**
  - Therefore, to prepare a dose of 300mg IBUPROFEN
- **Provide numrical answer**
  - 300mg 100mg/5ml = 15ml
- **Specific dose**
  - Therefore, you need to give 15 ml from the bottle!
